# Epigenetic features in the oyster *Crassostrea gigas* suggestive of functionally relevant promoter DNA methylation in invertebrates

**DOI:** 10.3389/fphys.2014.00129

**Published:** 2014-04-07

**Authors:** Guillaume Rivière

**Affiliations:** ^1^Institute for Fundamental and Applied Biology, Normandy UniversityCaen, France; ^2^UMR BOREA ‘Biologie des Organismes et Ecosystèmes Aquatiques’ Université de Caen Basse-Normandie, MNHN, UPMC, CNRS-7208, IRD-207Caen, France

**Keywords:** DNA methylation, transcription, oyster, invertebrates, functional epigenomics, evolution, lophotrochozoans, promoter

## Abstract

DNA methylation is evolutionarily conserved. Vertebrates exhibit high, widespread DNA methylation whereas invertebrate genomes are less methylated, predominantly within gene bodies. DNA methylation in invertebrates is associated with transcription level, alternative splicing, and genome evolution, but functional outcomes of DNA methylation remain poorly described in lophotrochozoans. Recent genome-wide approaches improve understanding in distant taxa such as molluscs, where the phylogenetic position, and life traits of *Crassostrea gigas* make this bivalve an ideal model to study the physiological and evolutionary implications of DNA methylation. We review the literature about DNA methylation in invertebrates and focus on DNA methylation features in the oyster. Indeed, though our MeDIP-seq results confirm predominant intragenic methylation, the profiles depend on the oyster's developmental and reproductive stage. We discuss the perspective that oyster DNA methylation could be biased toward the 5′-end of some genes, depending on physiological status, suggesting important functional outcomes of putative promoter methylation from cell differentiation during early development to sustained adaptation of the species to the environment.

DNA methylation is an epigenetic mechanism of great biological significance which is widely conserved in evolution (Zemach et al., 2010). DNA methylation in prokaryotes mostly affects adenines and is implicated in a broad range of processes from the control of DNA replication (review in Collier, 2009) to the defense against bacteriophages (Zaleski et al., 2005). Besides, bacterial DNA methylation contributes to the inheritable control of gene expression, maintaining phenotypes through cell generations, as observed for virulence genes (Heusipp et al., 2007).

In contrast, DNA in eukaryote genomes is predominantly methylated on cytosines (Zemach et al., 2010). However, significant differences exist between kingdoms. In animal genomes cytosines are methylated mostly within CpG dinucleotides (Doskočil and Šorm, 1962; Lister et al., 2009), whereas plants (Cokus et al., 2008), and fungi (Antequera et al., 1985; Selker and Stevens, 1987) can also have methylcytosines in the CHG or CHH contexts. In addition, genomes display markedly different methylation profiles between vertebrates and invertebrates. Vertebrates exhibit a high DNA methylation, whereas invertebrate genomes are far less methylated (Suzuki et al., 2007; Suzuki and Bird, 2008; Feng et al., 2010; Zemach et al., 2010).

In vertebrates, DNA methylation affects the whole genome, but eventually drops in the 5′ regions of genes (Zemach et al., 2010) which divide into two groups regarding their CpG content (Antequera, 2003). The low-CpG promoters are hypermethylated and drive the transcription of tissue-specific genes. Instead, the high-CpG promoters where CG dinucleotide-rich regions define “CpG islands” (CGIs) are hypomethylated and control widely expressed genes (Elango and Yi, 2008). Depending on their density, methylcytosines in promoters restrict the access of the transcription machinery to transcription start sites due to CpG-binding proteins recruitment (Antequera, 2003; Deaton et al., 2011; Vinson and Chatterjee, 2012) and/or the regulation of DNA/histone interactions within nucleosomes (Cedar and Bergman, 2009; Zentner and Henikoff, 2013) thereby diminishing transcription (Hsieh, 1994; Rivière et al., 2011).

Invertebrates generally display a heterogenic or “mosaic” methylation profile with predominant methylation of transcription units (Suzuki et al., 2007; Elango et al., 2009; Gavery and Roberts, 2010; Walsh et al., 2010; Gadau et al., 2012; Sarda et al., 2012). High-throughput sequencing technologies have rapidly increased our knowledge on DNA methylation in ecdysozoans (encompassing nematodes and arthropods), especially insects (Lyko et al., 2000; Elango et al., 2009; Park et al., 2011; Zwier et al., 2012), bringing new insights into the evolution of DNA methylation functions. Now, these questions have to be addressed in more distant invertebrate taxa such as lophotrochozoans (encompassing worms and molluscs), which remain widely underdescribed. Indeed, despite methylated DNA being present in the snail *Biomphalaria glabrata* (Fneich et al., 2013), the scallops *Chlamys farreri* and *Patinopecten yessoensis* (Wang et al., 2008), the truncated wedgeshell *Donax trunculus* (Petrovic et al., 2009) and the pacific oyster *Crassostrea gigas* (Gavery and Roberts, 2010; Riviere et al., 2013), only one single-base resolution methylome is available to date in a mollusc, the pacific oyster *Crassostrea gigas* (Gavery and Roberts, 2013).

*C. gigas* represents an interesting species for the study of DNA methylation in lophotrochozoans, with regards to its peculiar life traits, economic and ecological importance. Indeed, *C. gigas*, undergoes a pelagic development and metamorphoses before a benthic adult phase in the highly stressful intertidal area. Besides, oysters are successive hermaphrodites due to a yearly gonad renewal from stem cells. Therefore, the entire life cycle of oysters is punctuated with dramatic morpho-physiological changes, which rely on the implementation of transient transcriptomes within changing environments. The control of these transcriptomes likely implicates epigenetic mechanisms, which remain to be elucidated. The recent characterization of the *C. gigas* genome (Zhang et al., 2012) enabled an assessment that DNA methylation was mostly intragenic in the oyster (Gavery and Roberts, 2013). However, our recent results indicate that oyster methylation patterns display temporal variations and could be uniquely biased toward the 5′-upstream region of gene subsets depending on physiological contexts (Riviere et al., 2013). Therefore, in this work we first review the literature on invertebrate DNA methylation focusing on gene body methylation (GBM) and highlight the current knowledge in the oyster. Then, we discuss the perspective of functional outcomes of possible specific DNA methylation features in *C. gigas*.

## DNA methylation in invertebrates

### Evolution of methylation patterns in invertebrates

The prevalence of DNA methylation within gene bodies in most invertebrates studied to date suggests that GBM is the ancestral state of this phenomenon (Sarda et al., 2012). Because methylated cytosines tend to spontaneously deaminate into thymines, an underrepresentation of the CpG dinucleotide in a region of interest reflects a sustained methylation (Bird, 1980). Therefore, the methylation level of genes is often inferred from the “normalized CpG content” (CpG O/E ratio), which compares the observed to the expected CpG dinucleotide contents (Shimizu et al., 1997). Several studies (Suzuki et al., 2007; Elango et al., 2009; Gavery and Roberts, 2010) confirmed the robust negative correlation between the methylation level in the germline, i.e., possibly inherited across generations, and the CpG O/E ratio. Because of the increased mutation rate of methylated cytosines, DNA methylation is considered important in genome evolution. Accordingly, distant lineages present different patterns of GBM (Zemach et al., 2010; Sarda et al., 2012). Among divergent invertebrate groups, Sarda and colleagues reported that ecdysozoans (i.e., the honeybee *Apis mellifera* and the silkworm *Bombyx mori*) exhibit a lower GBM than the cnidarian anemone *Nematostella vectensis* and the tunicate sea squirt *Ciona intestinalis* (Sarda et al., 2012). This indicates that protostomes (encompassing ecdysozoans and lophotrochozoans), have evolved toward a loss of (gene body) methylation, when compared to deuterostome animals (encompassing vertebrates) and to their common ancestor. The localization of methylated cytosines within transcription units also displays lineage-specific differences. Insects tend to have a higher methylation of the 5′ than of the 3′ regions of genes, whereas these levels are not different in the sea squirt, whilst their common ancestor, represented by the sea anemone, shows an intermediate profile (Zemach et al., 2010; Wang et al., 2013). The distinct evolutionary histories of GBM between taxa are further illustrated by the relationship between methylation and transposable elements (TE). Indeed, in contrast to vertebrates (Yoder et al., 1997), methylation is clearly not the main mechanism used by insects in order to silence the genes lying within TEs (Lyko et al., 2010; Wang et al., 2013), although ants demonstrate a high TE methylation (Bonasio et al., 2012).

### The role of methylation in invertebrate genome evolution

A general association between methylation of transcription units and protein conservation in invertebrates emerges from studies on insects (Hunt et al., 2010; Wang et al., 2013), cnidarian, and tunicates (Zemach et al., 2010; Flores et al., 2012; Sarda et al., 2012). Indeed, despite slight particular features in the pea aphid (Hunt et al., 2010), highly methylated genes have a higher number of orthologs than sparsely methylated genes (Sarda et al., 2012). This is surprising because the hyper-mutability of methylcytosines would be expected to make DNA sequences diverge over evolutionary time (Cooper and Krawczak, 1989; Elango et al., 2008; Chuang and Chen, 2014). However, the weak cytosine representation within low CpG O/E genes could result in a lower frequency of methylation-dependent mutations (Hunt et al., 2010). Besides, mutations within genes methylated across many generations (low CpG O/E) are likely deleterious and thus would not be sustained in the genome.

### Relationship between DNA methylation and transcript variants in invertebrates

Despite distinct evolution histories between taxa, the role of GBM in transcript variant selection seems to be conserved. For example, GBM is widely implicated in the generation of alternative transcripts in mammals (Maunakea et al., 2010, 2013). Likewise, exon methylation is inversely correlated to exon skipping, and alternative splicing is increased among methylated genes in the honeybee (Flores et al., 2012). Consistently, *Nasonia* exons are “tagged” with cytosine methylation whereas introns are mostly unmethylated (Wang et al., 2013). Nevertheless, such patterns are not mandatory for a role of GBM in exon skipping. Indeed, *Nasonia* differentially spliced genes do not have an increased methylation probability, and methylated and non-methylated genes do not differ in their degree of alternative splicing (Wang et al., 2013). In the oyster, exons are preferentially methylated, albeit introns also exhibit significant methylation (Gavery and Roberts, 2013). Therefore, caution should be taken when inferring the role of methylation in exon selection from phylogenetic proximity and/or methylation pattern similarity between species.

### GBM in invertebrates is associated with transcriptional regulation

A greater number of transcripts can arise from methylated genes than from unmethylated genes in insects, possibly because methylation influences exon inclusion during transcription (Flores et al., 2012). In the oyster, a low inherited GBM (high CpG O/E) is supposed to increase the “transcriptional opportunities.” This refers to the association of DNA methylation with biases in the binding of the transcription proteins, thereby increasing the number of transcript variants (Roberts and Gavery, 2012). Thus, GBM would provide a mechanism for enhanced phenotypic plasticity from a limited number of genes. Parallel to transcript variant selection, the conservation of GBM also favors its biological significance in the regulation of transcription levels. In vertebrates, in contrast to promoter methylation which generally represses transcription (Bird, 1992; Deaton et al., 2011; Vinson and Chatterjee, 2012), GBM is associated with active transcription (Ball et al., 2009; Maunakea et al., 2010). In invertebrates which are mostly considered devoid of significant promoter CpG methylation (Elango and Yi, 2008), GBM also influences mRNA expression depending on the density of methylation (Feng et al., 2010; Xiang et al., 2010; Zemach et al., 2010). Genome-wide observations across invertebrates indicate that transcriptional units are either highly or slightly methylated, and thereby display a general bimodal distribution regarding methylation. Nonetheless, the relationship between methylation and transcription exhibit species-specific features. Indeed, a positive linear correlation between GBM and mRNA levels exists in *Nematostella vectensis* and *Bombyx mori* (Xiang et al., 2010; Zemach et al., 2010). By contrast, in the honeybee most genes that are either highly or weakly expressed are heavily methylated (Zemach et al., 2010). In adult oyster gills, transcript levels increase with GBM until the 40th percentile and remain stable until the 100th percentile where methylation decreases (Gavery and Roberts, 2013).

It is remarkable that gene subsets with distinct methylation levels correspond to distinct functions. In insects, genes with high canonical methylation (low CpG O/E) tend to be constitutively expressed at moderate to high levels and related to housekeeping functions, a conserved trend in the distant invertebrates investigated (Elango et al., 2009; Hunt et al., 2010; Sarda et al., 2012). Conversely, genes exhibiting little inherited methylation (high CpG O/E) display a broader range of mRNA levels and are implicated in regulated and/or inducible functions such as cell signaling and environmental stimulus response (Hunt et al., 2010). Accordingly, environmentally-induced methylomes control caste-related phenotypes in ants and honeybees (Lyko et al., 2010; Bonasio et al., 2012). In molluscs, similar interpretations were inferred from CpG O/E ratios (Gavery and Roberts, 2010; Fneich et al., 2013), further supported by the direct influence of DNA methylation on the transcription of development genes in the oyster (Riviere et al., 2013).

## Perspective: are there unique DNA methylation features in oysters with functional consequences?

In molluscs, cytosines are more methylated than in other invertebrate taxa (ca. 2 vs. ca. 0.15% in insects) (Zemach et al., 2010; Fneich et al., 2013; Gavery and Roberts, 2013). This indicates that molluscs exhibit specific characteristics in DNA methylation, suggesting more subtle roles and functional outcomes for this epigenetic mark than originally considered.

### Is there a “CGI promoter-like” transcriptional regulation in oysters?

**Figure 1:**
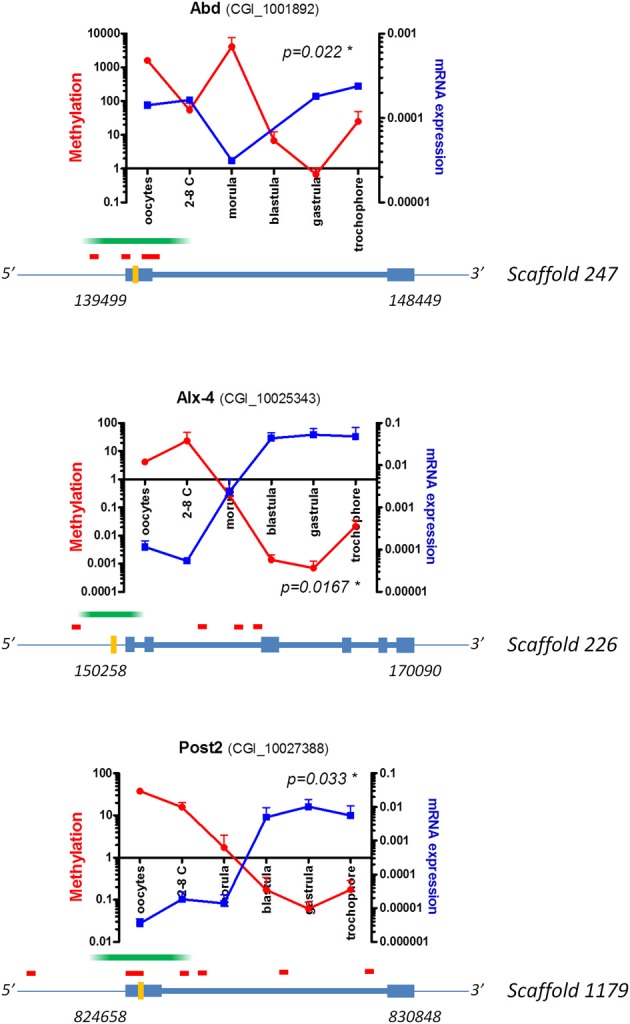
**The methylation of 5′ regions can drive transcriptional repression during oyster development.** Examples of development genes showing patterns suggestive of DNA methylation bias in upstream domains. DNA methylation (red) investigated by MeDIP-qPCR and cognate mRNA expression (blue) measured by RT-qPCR are shown for the *C. gigas Abd*, *Alx4* and *Post2* putative orthologs (development stages and GenBank accession numbers are indicated). The diagrams represent the genomic and methodological contexts. Scaffold numbers correspond to the fragment considered in the present assembly of the oyster genome (Zhang et al., 2012), start and stop positions (italic); intergenic (thin line), introns (thick line), exons (rectangles), putative MeDIPped fragments (green), qPCR amplified regions (orange), and CpG islands (red) are represented (modified from Riviere et al., 2013). ^*^p < 0.05 Pearson or Spearman's correlation test between methylation and mRNA expression.

Intriguingly, a negative correlation exists between the mRNA level and the specific methylation of some oyster development genes (Riviere et al., 2013). This apparently contradicts the association of invertebrate GBM with active transcription (Ball et al., 2009; Maunakea et al., 2010; Xiang et al., 2010; Zemach et al., 2010; Gavery and Roberts, 2013), except if one considers that the methylated regions could extend upstream of gene bodies to the 5′ domain. Interestingly, such regions corresponding to the predicted proximal promoter and/or first exon exhibit CpG islands (CGIs) (Figure 1). Due to the limitations in the MeDIP-qPCR methodology used, it remains unclear which of these regions are the actual targets of methylation. However, the recent single-base resolution methylome of adult *C. gigas* gills (Gavery and Roberts, 2013) reveals a significant methylation of putative promoter regions. Together, these observations suggest a bias in DNA methylation toward the 5′ region of genes, which diminishes transcription at least for some genes at defined physiological states in the oyster. Therefore, we hypothesize that a “CGI promoter-like” transcriptional regulation could exist in *C. gigas*, in contrast with general findings in invertebrates (Elango and Yi, 2008; Zemach et al., 2010; Sarda et al., 2012), but in line with the results concerning the aplysia CREB2 gene (Rajasethupathy et al., 2012).

### Do dynamic changes in DNA methylation underly physiological plasticity?

**Figure 2:**
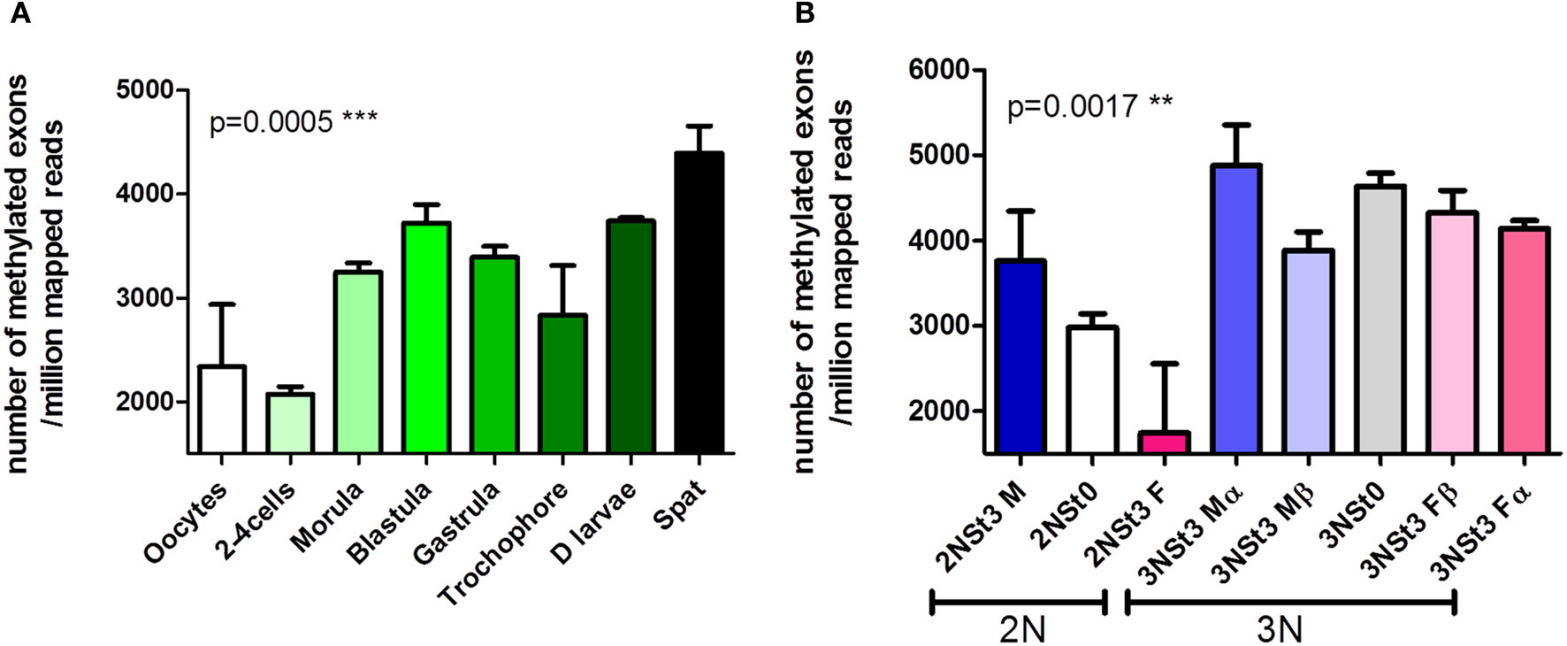
**Dynamic changes in the exome-wide methylation pattern during the oyster development (A) and gametogenesis (B).** Exon mapping after MeDIP-seq at the different stages indicated. Values are given as the mean ± s.e.m. of triplicate experiments. *P* values are given for One-Way ANOVA (*p* < 0.05 was considered significant). Development and gametogenesis stages are indicated. F, female; M, male; St0, sexual resting; St3, mature gametes; α, alpha fertile phenotype; β, beta sterile phenotype (Riviere et al., in preparation). ^**^p < 0.01; ^***^p < 0.001; One-way ANOVA for number of methylated exons/million mapped reads.

Consistent with variations in the amount of cytosine methylation during early oyster development (Riviere et al., 2013), our recent MeDIP-seq analyses indicate that developmental stages differ not only in the number of methylated genes, but also in the number of methylated regions per gene (Figure 2A). In adults, additional information comes from the study of the gametogenesis. Indeed, the methylation profiles are different between sexual resting (stage 0) and maturity (stage 3) as well as between males and females (Figure 2B) (Riviere et al., in preparation). Triploid oysters were originally thought to be sterile because of aneuploidy-induced blocking of the gonial mitosis (“beta” triploids). However, some animals (“alpha” triploids) can escape sterility and produce mature gametes (Jouaux et al., 2010). Interestingly, methylation patterns in fertile alpha triploids are significantly different from those of sterile beta animals, yet are similar to those of fertile diploid oysters (Figure 2B). We found that specific methylation profiles are dynamic and match specific embryonic and germinal phenotypes in oysters. Hence, the association between DNA methylation and the regulation of differentiation genes in stem cells in mammals (review in Jones, 2012). Therefore, we assume a central role for DNA methylation in the differentiation of stem cells in *C. gigas*.

Considering gene expression as a fundamentally random, stochastic phenomenon (Laforge et al., 2005; Raj and van Oudenaarden, 2008; Kupiec, 2010), DNA methylation would bias the number of probabilities in gene transcription in a given cellular context. As a consequence, methylomes would underlie transcriptomes at the cellular level. A consistent hypothesis proposed GBM to increase the possibility of transcript variants from genes with low germline methylation, thereby underlying the phenotypic plasticity that allows *C. gigas* to adapt to unpredictable conditions at the population level (Roberts and Gavery, 2012). Therefore, the high CpG O/E ratio of oyster development genes, corresponding to an “inducible” profile, is inconsistent with their determined transcriptional patterns required in the context of a “fixed development program.” In contrast, it rather fits with the present assumption that the development of *C. gigas* relies on the environmental stabilization of stochastic transcriptomes through epigenetic mechanisms like DNA methylation. Our hypothesis is supported by both the variation in the amount of methyl-DNA during embryogenesis and the suspected presence of promoter DNA methylation influencing oyster developmental gene transcription (Riviere et al., 2013). From these observations which broaden former hypotheses (Roberts and Gavery, 2012; Gavery and Roberts, 2014), we propose that DNA methylation bears a crucial role in most if not all physiological processes which implicate the implementation and the dynamic regulation of transient transcriptomes throughout the oyster life cycle.

### Does DNA methylation in the oyster bridge the gap between cell differentiation, environmental adaptation, and evolutionary success?

It remains to be elucidated whether methylation governs transcription, whether it just reflects local transcriptionally-active open chromatin, or both. Regardless, we assume that dynamic genome-wide DNA methylation profiles are integrated through the canalization of random interactions with the surrounding molecular machinery in the chromatin context (Badeaux and Shi, 2013) and lead to the implementation of environmentally-induced transcriptomes. Successful transcriptomes enable the development of sexually mature animals that may imprint methyl marks to DNA into their germinal cells, thereby passing successful methylomes to their progeny. If repeated across oyster generations, such successful methylomes could induce mutations and permanent changes in the genome. Therefore, in oysters DNA methylation could constitute a pivotal mechanism underlying processes from (i) cell differentiation during embryogenesis, (ii) organism survival in changing environments (Vandegehuchte and Janssen, 2013), and (iii) ultimately species adaptation over evolutionary time (Keller and Taylor, 2008). This confers a major role for DNA methylation in cell Darwinism and ontophylogenesis in oysters (Maresca and Schwartz, 2006; Rollo, 2006). However, one should remain cautious with this hypothesis because transgenerational imprinting has, to our knowledge, never been demonstrated in lophotrochozoans.

## Future directions in DNA methylation research in oysters

The possible relevance of promoter methylation has been neglected in most invertebrates noticeably because of weak CpG representation and/or methylation in promoter regions (Elango and Yi, 2008). However, the transcriptional repression of the *Aplysia* CREB2 gene by the methylation of a CpG island in the promoter (Rajasethupathy et al., 2012) parallels our finding that 5′-methylation and mRNA expression can be negatively correlated (Riviere et al., 2013). Such a “CGI promoter-like” regulation in invertebrates remains to be investigated, and *in vitro* methylated reporter gene constructs might be helpful.

Current genome-wide studies bring informative static pictures of invertebrate methylomes. Forthcoming investigations should benefit from high-throughput technologies to decipher the dynamics of such methylomes and the mechanisms underlying their functional implications. Future research should also address the complete epigenetic landscape of the chromatin beside cytosine methylation, including nucleosome occupancy and histone marks. Indeed, in flies (Ebert et al., 2006) and mammals (Rach et al., 2011) similar DNA methylation levels have different outcomes, depending on the chromatin context. Furthermore, histone modifications are potentially relevant in oyster development and reproduction (Fellous et al., 2014).

How the environment induces changes in DNA methylation, especially in physiological contexts like development and reproduction, and whether these changes are persistent are also major issues. Indeed, as in mammals (Gabory et al., 2011), diet inputs during larval life control the expression of caste-related gene subsets in the honeybee (Kucharski et al., 2008). Moreover, investigations in *Daphnia magna* (Vandegehuchte et al., 2009, 2010) are suggestive of the trans-generational persistence of environmentally-induced epigenetic modifications in arthropods. Such “imprinting” mechanisms and the implication of the DNA methylation within remain to be demonstrated in lophotrochozoans, especially in “invasive” species like *C. gigas*. In this context, the estimated low inherited methylation of “inducible” oyster genes (Gavery and Roberts, 2010) suggests a possible reversibility of DNA methylation which remains to be explored in invertebrates.

These questions open a major field of research and warrant in-depth studies in lophotrochozoans, which encompass a tremendous variability of species and life traits. Many different reasons support the oyster as a model of interest in this perspective: their peculiar ecology, the availability of their genome, the development of functional tools, the worldwide distribution of the species, its economic importance, and the present perspective of functionally relevant specific DNA methylation features. A better knowledge of the dynamics, interactions, and roles of DNA methylation in *Crassostrea gigas* could greatly improve our understanding of this epigenetic mark in invertebrates.

### Conflict of interest statement

The author declares that the research was conducted in the absence of any commercial or financial relationships that could be construed as a potential conflict of interest.

## References

[B1] Antequera F. (2003). Structure, function and evolution of CpG island promoters. Cell. Mol. Life Sci. 60, 1647–1658 10.1007/s00018-003-3088-6 14504655 PMC11138798

[B2] Antequera F. Tamame M. Vilanueva J. R. Santos T. (1985). Developmental modulation of DNA methylation in the fungus Phycomyces blakesleeanus. Nucleic Acids Res. 13, 6545–6558 10.1093/nar/13.18.6545 2997714 PMC321976

[B3] Badeaux A. I. Shi Y. (2013). Emerging roles for chromatin as a signal integration and storage platform. Nat. Rev. Mol. Cell Biol. 14, 211–224 10.1038/nrm3545 23847782

[B4] Ball M. P. Li J. B. Gao Y. Lee J. Leproust E. M. Park I. (2009). Targeted and genome-scale strategies reveal gene-body methylation signatures in human cells. Nat. Biotechnol. 27, 361–369 10.1038/nbt.1533 19329998 PMC3566772

[B5] Bird A. (1992). The Essentials of DNA methylation Minireview. Cell 70, 5–8 10.1016/0092-8674(92)90526-I 1377983

[B6] Bird A. P. (1980). DNA methylation and the frequency of CpG in animal DNA. Nucleic Acid Res. 8, 1499–1504 10.1093/nar/8.7.1499 6253938 PMC324012

[B7] Bonasio R. Li Q. Lian J. Mutti N. S. Jin L. Zhao H. (2012). Genome-wide and caste-specific DNA methylomes of the ants Camponotus floridanus and Harpegnathos saltator. Curr. Biol. 22, 1755–1764 10.1016/j.cub.2012.07.042 22885060 PMC3498763

[B8] Cedar H. Bergman Y. (2009). Linking DNA methylation and histone modification: patterns and paradigms. Nat. Rev. Genet. 10, 295–304 10.1038/nrg2540 19308066

[B9] Chuang T. J. Chen F. C. (2014). DNA methylation is associated with an increased level of conservation at nondegenerate nucleotides in mammals. Mol. Biol. Evol. 31, 387–396 10.1093/molbev/mst208 24157417 PMC3907051

[B10] Cokus S. J. Feng S. Zhang X. Chen Z. Merriman B. Haudenschild C. D. (2008). Shotgun bisulphite sequencing of the Arabidopsis genome reveals DNA methylation patterning. Nature 452, 215–219 10.1038/nature06745 18278030 PMC2377394

[B11] Collier J. (2009). Epigenetic regulation of the bacterial cell cycle. Curr. Opin. Microbiol. 12, 722–729 10.1016/j.mib.2009.08.005 19783470

[B12] Cooper D. N. Krawczak M. (1989). Cytosine methylation and the fate of CpG dinucleotides in vertebrate genomes. Hum. Genet. 83, 181–188 10.1007/BF00286715 2777259

[B13] Deaton A. M. Bird A. Deaton M. (2011). CpG islands and the regulation of transcription. Genes Dev. 25, 1010–1022 10.1101/gad.2037511 21576262 PMC3093116

[B14] Doskočil J. Šorm F. (1962). Distribution of 5-methylcytosine in pyrimidine sequences of deoxyribonucleic acids. Biochim. Biophys. Acta 55, 953–959 10.1016/0006-3002(62)90909-5 13887466

[B15] Ebert A. Lein S. Schotta G. Reuter G. (2006). Histone modification and the control of heterochromatic gene silencing in Drosophila. Chromosome Res. 14, 377–392 10.1007/s10577-006-1066-1 16821134

[B16] Elango N. Hunt B. G. Goodisman M. A. D. Yi S. V. (2009). DNA methylation is widespread and associated with differential gene expression in castes of the honeybee, Apis mellifera. Proc. Natl. Acad. Sci. U.S.A. 106, 11206–11211 10.1073/pnas.0900301106 19556545 PMC2708677

[B17] Elango N. Kim S.-H. Vigoda E. Yi S. V. (2008). Mutations of different molecular origins exhibit contrasting patterns of regional substitution rate variation. PLoS Comput. Biol. 4:e1000015 10.1371/journal.pcbi.1000015 18463707 PMC2265638

[B18] Elango N. Yi S. V. (2008). DNA methylation and structural and functional bimodality of vertebrate promoters. Mol. Biol. Evol. 25, 1602–1608 10.1093/molbev/msn110 18469331

[B19] Fellous A. Favrel P. Guo X. Riviere G. (2014). The Jumonji gene family in *Crassostrea gigas* suggests evolutionary conservation of Jmj-C histone demethylases orthologues in the oyster gametogenesis and development. Gene 538, 164–175 10.1016/j.gene.2013.12.016 24406622

[B20] Feng S. Cokus S. J. Zhang X. Chen P. Bostick M. Goll M. G. (2010). Conservation and divergence of methylation patterning in plants and animals. Proc. Natl. Acad. Sci. U.S.A. 107, 8689–8694 10.1073/pnas.1002720107 20395551 PMC2889301

[B21] Flores K. Wolschin F. Corneveaux J. J. Allen A. N. Huentelman M. J. Amdam G. V. (2012). Genome-wide association between DNA methylation and alternative splicing in an invertebrate. BMC Genomics 13:480 10.1186/1471-2164-13-480 22978521 PMC3526459

[B22] Fneich S. Dheilly N. Adema C. Rognon A. Reichelt M. Bulla J. (2013). 5-methyl-cytosine and 5-hydroxy-methyl-cytosine in the genome of *Biomphalaria glabrata*, a snail intermediate host of Schistosoma mansoni. Parasit. Vectors 6, 167–178 10.1186/1756-3305-6-167 23742053 PMC3681652

[B23] Gabory A. Attig L. Junien C. (2011). Epigenetic mechanisms involved in developmental nutritional programming. World J. Diabetes 2, 164–175 10.4239/wjd.v2.i10.164 22010058 PMC3196195

[B24] Gadau J. Helmkampf M. Nygaard S. Roux J. Simola D. F. (2012). The genomic impact of 100 million years of social evolution in seven ant species. Trends Genet. 28, 14–21 10.1016/j.tig.2011.08.005 21982512 PMC3314025

[B25] Gavery M. R. Roberts S. B. (2010). DNA methylation patterns provide insight into epigenetic regulation in the Pacific oyster (*Crassostrea gigas*). BMC Genomics 11:483 10.1186/1471-2164-11-483 20799955 PMC2996979

[B26] Gavery M. R. Roberts S. B. (2013). Predominant intragenic methylation is associated with gene expression characteristics in a bivalve mollusc. PeerJ 1:e215 10.7717/peerj.215 24282674 PMC3840415

[B27] Gavery M. R. Roberts S. B. (2014). A context dependent role for DNA methylation in bivalves. Brief. Funct. Genomics. [Epub ahead of print]. 10.1093/bfgp/elt054 24397979

[B28] Heusipp G. Fälker S. Schmidt M. A. (2007). DNA adenine methylation and bacterial pathogenesis. Int. J. Med. Microbiol. 297, 1–7 10.1016/j.ijmm.2006.10.002 17126598

[B29] Hsieh C. (1994). Dependence of transcriptional repression on CpG methylation density. Mol. Cell. Biol. 14, 5487–5494 7518564 10.1128/mcb.14.8.5487-5494.1994 PMC359068

[B30] Hunt B. G. Brisson J. A. Yi S. V Goodisman M. A. D. (2010). Functional Conservation of DNA methylation in the Pea Aphid and the Honeybee. Genome Biol. Evol. 2, 719–728 10.1093/gbe/evq057 20855427 PMC2962555

[B31] Jones P. A. (2012). Functions of DNA methylation: islands, start sites, gene bodies and beyond. Nat. Rev. Genet. 13, 484–492 10.1038/nrg3230 22641018

[B32] Jouaux A. Heude-Berthelin C. Sourdaine P. Mathieu M. Kellner K. (2010). Gametogenic stages in triploid oysters *Crassostrea gigas*: irregular locking of gonial proliferation and subsequent reproductive effort. J. Exp. Mar. Biol. Ecol. 395, 162–170 10.1016/j.jembe.2010.08.030

[B33] Keller S. R. Taylor D. R. (2008). History, chance and adaptation during biological invasion: separating stochastic phenotypic evolution from response to selection. Ecol. Lett. 11, 852–866 10.1111/j.1461-0248.2008.01188.x 18422638

[B34] Kucharski R. Maleszka J. Foret S. Maleszka R. (2008). Nutritional control of reproductive status in honeybees via DNA methylation. Science 319, 1827–1830 10.1126/science.1153069 18339900

[B35] Kupiec J. (2010). On the lack of speci fi city of proteins and its consequences for a theory of biological organization. Prog. Biophys. Mol. Biol. 102, 45–52 10.1016/j.pbiomolbio.2009.11.002 19917305

[B36] Laforge B. Guez D. Martinez M. Kupiec J.-J. (2005). Modeling embryogenesis and cancer: an approach based on an equilibrium between the autostabilization of stochastic gene expression and the interdependence of cells for proliferation. Prog. Biophys. Mol. Biol. 89, 93–120 10.1016/j.pbiomolbio.2004.11.004 15826673

[B37] Lister R. Pelizzola M. Dowen R. H. Hawkins R. D. Hon G. Tonti-filippini J. (2009). Human DNA methylomes at base resolution show widespread epigenomic differences. Nature 462, 315–322 10.1038/nature08514 19829295 PMC2857523

[B38] Lyko F. Foret S. Kucharski R. Wolf S. Falckenhayn C. Maleszka R. (2010). The honey bee epigenomes: differential methylation of brain DNA in queens and workers. PLoS Biol. 8:e1000506 10.1371/journal.pbio.1000506 21072239 PMC2970541

[B39] Lyko F. Ramsahoye B. H. Jaenisch R. (2000). DNA methylation in Drosophila melanogaster. Nature 408, 538–540 10.1038/35046205 11117732

[B40] Maresca B. Schwartz J. H. (2006). Sudden origins: a general mechanism of evolution based on stress protein concentration and rapid environmental change. Anat. Rec. B New Anat. 289, 38–46 10.1002/ar.b.20089 16437551

[B41] Maunakea A. K. Chepelev I. Cui K. Zhao K. (2013). Intragenic DNA methylation modulates alternative splicing by recruiting MeCP2 to promote exon recognition. Cell Res. 11, 1–14 10.1038/cr.2013.110 23938295 PMC3817542

[B42] Maunakea A. K. Nagarajan R. P. Bilenky M. Ballinger T. J. Souza C. D. Fouse S. D. (2010). Conserved role of intragenic DNA methylation in regulating alternative promoters. Nature 466, 253–257 10.1038/nature09165 20613842 PMC3998662

[B43] Park J. Peng Z. Zeng J. Elango N. Park T. Wheeler D. (2011). Comparative analyses of DNA methylation and sequence evolution using Nasonia genomes. Mol. Biol. Evol. 28, 3345–3354 10.1093/molbev/msr168 21693438 PMC3215512

[B44] Petrovic V. Perez-Garcia C. Pasantes J. Satovic E. Prats E. Plohl M. (2009). A GC-rich satellite DNA and karyology of the bivalve mollusk *Donax trunculus*: a dominance of GC-rich heterochromatin. Cytogenet. Genome Res. 124, 63–71 10.1159/000200089 19372670

[B45] Rach E. A. Winter D. R. Benjamin A. M. Corcoran D. L. Ni T. (2011). Transcription initiation patterns indicate divergent strategies for gene regulation at the chromatin level. PLoS Genet. 7:e100127 10.1371/journal.pgen.1001274 21249180 PMC3020932

[B46] Raj A. van Oudenaarden A. (2008). Nature, nurture, or chance: stochastic gene expression and its consequences. Cell 135, 216–226 10.1016/j.cell.2008.09.050 18957198 PMC3118044

[B47] Rajasethupathy P. Antonov I. Sheridan R. Frey S. Sander C. Tuschl T. (2012). A role for neuronal piRNAs in the epigenetic control of memory-related synaptic plasticity. Cell 149, 693–707 10.1016/j.cell.2012.02.057 22541438 PMC3442366

[B48] Rivière G. Lienhard D. Andrieu T. Vieau D. Frey B. M. Frey F. J. (2011). Epigenetic regulation of somatic angiotensin-converting enzyme by DNA methylation and histone acetylation. Epigenetics 6, 478–489 10.4161/epi.6.4.14961 21364323

[B49] Riviere G. Wu G. Fellous A. Goux D. Sourdaine P. Favrel P. (2013). DNA methylation is crucial for the early development in the Oyster *C. gigas*. Mar. Biotechnol. 15, 739–753 10.1007/s10126-013-9523-2 23877618

[B50] Roberts S. B. Gavery M. R. (2012). Is there a relationship between DNA methylation and phenotypic plasticity in invertebrates? Front. Physiol. 2:116 10.3389/fphys.2011.00116 22232607 PMC3249382

[B51] Rollo C. D. (2006). Radiation and the regulatory landscape of neo2-Darwinism. Mutat. Res. 597, 18–31 10.1016/j.mrfmmm.2005.09.009 16414092

[B52] Sarda S. Zeng J. Hunt B. G. Yi S. V. (2012). The evolution of invertebrate gene body methylation. Mol. Biol. Evol. 29, 1907–1916 10.1093/molbev/mss062 22328716

[B53] Selker E. U. Stevens J. N. (1987). Signal for DNA methylation associated with tandem duplication in Neurospora crassa. Mol. Cell. Biol. 7, 1032–1038 2951588 10.1128/mcb.7.3.1032-1038.1987 PMC365173

[B54] Shimizu T. S. Takahashi K. Tomita M. (1997). CpG distribution patterns in methylated and non-methylated species. Gene 205, 103–107 10.1016/S0378-1119(97)00542-8 9461383

[B55] Suzuki M. M. Bird A. (2008). DNA methylation landscapes: provocative insights from epigenomics. Nat. Rev. Genet. 9, 465–476 10.1038/nrg2341 18463664

[B56] Suzuki M. M. Kerr A. R. W. De Sousa D. Bird A. (2007). CpG methylation is targeted to transcription units in an invertebrate genome. Genome Res. 17, 625–631 10.1101/gr.6163007 17420183 PMC1855171

[B57] Vandegehuchte M. B. Janssen C. R. (2013). Epigenetics in an ecotoxicological context. Mutat. Res. [Epub ahead of print]. 10.1016/j.mrgentox.2013.08.008 24004878

[B58] Vandegehuchte M. B. Lemière F. Janssen C. R. (2009). Quantitative DNA-methylation in *Daphnia magna* and effects of multigeneration Zn exposure. Comp. Biochem. Physiol. C Toxicol. Pharmacol. 150, 343–348 10.1016/j.cbpc.2009.05.014 19486948

[B59] Vandegehuchte M. B. Lemière F. Vanhaecke L. Vanden Berghe W. Janssen C. R. (2010). Direct and transgenerational impact on *Daphnia magna* of chemicals with a known effect on DNA methylation. Comp. Biochem. Physiol. C Toxicol. Pharmacol. 151, 278–285 10.1016/j.cbpc.2009.11.007 19961956

[B60] Vinson C. Chatterjee R. (2012). CG methylation. Future Med. 4, 655–663 10.2217/epi.12.55 23244310 PMC3566568

[B61] Walsh T. K. Brisson J. A. Robertson H. M. Gordon K. Jobert-Possamai S. Tagu D. (2010). A functional DNA methylation system in the pea aphid, Acyrthosiphon pisum. Insect Mol. Biol. 19, 215–228 10.1111/j.1365-2583.2009.00974.x 20482652

[B62] Wang S. Bao Z. Hu X. Shao M. Zhang L. Hu J. (2008). Two novel elements (CFG1 and PYG1) of Mag lineage of Ty3/Gypsy retrotransposons from Zhikong scallop (*Chlamys farreri*) and Japanese scallop (*Patinopecten yessoensis*). Genetica 133, 37–46 10.1007/s10709-007-9180-3 17694394

[B63] Wang X. Wheeler D. Avery A. Rago A. Choi J.-H. Colbourne J. K. (2013). Function and evolution of DNA methylation in Nasonia vitripennis. PLoS Genet. 9:e1003872 10.1371/journal.pgen.1003872 24130511 PMC3794928

[B64] Xiang H. Zhu J. Chen Q. Dai F. Li X. Li M. (2010). Single base-resolution methylome of the silkworm reveals a sparse epigenomic map. Nat. Biotechnol. 28, 516–520 10.1038/nbt.1626 20436463

[B65] Yoder J. A. Walsh C. P. Bestor T. H. (1997). Cytosine methylation and the ecology of intragenomic parasites. Trends Genet. 13, 335–340 10.1016/S0168-9525(97)01181-5 9260521

[B66] Zaleski P. Wojciechowski M. Piekarowicz A. (2005). The role of Dam methylation in phase variation of Haemophilus influenzae genes involved in defence against phage infection. Microbiology 151, 3361–3369 10.1099/mic.0.28184-0 16207918

[B67] Zemach A. McDaniel I. E. Silva P. Zilberman D. (2010). Genome-wide evolutionary analysis of eukaryotic DNA methylation. Science 328, 916–919 10.1126/science.1186366 20395474

[B68] Zentner G. E. Henikoff S. (2013). REVIEW Regulation of nucleosome dynamics by histone modifications. Nat. Struct. Mol. Biol. 20, 259–266 10.1038/nsmb.2470 23463310

[B69] Zhang G. Fang X. Guo X. Li L. Luo R. Xu F. (2012). The oyster genome reveals stress adaptation and complexity of shell formation. Nature 490, 49–54 10.1038/nature11413 22992520

[B70] Zwier M. V Verhulst E. C. Zwahlen R. D. Beukeboom L. W. van de Zande L. (2012). DNA methylation plays a crucial role during early Nasonia development. Insect Mol. Biol. 21, 129–138 10.1111/j.1365-2583.2011.01121.x 22122805

